# Follow-up and tracing of tuberculosis patients who fail to attend their scheduled appointments in Cotonou, Benin: a retrospective cohort study

**DOI:** 10.1186/s12913-015-1219-z

**Published:** 2016-01-11

**Authors:** Serge Ade, Arnaud Trébucq, Anthony D. Harries, Gabriel Ade, Gildas Agodokpessi, Prudence Wachinou, Dissou Affolabi, Sévérin Anagonou

**Affiliations:** 1 National Tuberculosis Programme, Cotonou, Benin; 2 Faculté de Médecine, Université de Parakou, Parakou, Benin; 3 International Union against Tuberculosis and Lung Disease, Paris, France; 4 London School of Hygiene and Tropical Medicine, London, UK

**Keywords:** Tuberculosis, Treatment, Scheduled appointment, Benin, Operational research

## Abstract

**Background:**

In the “Centre National Hospitalier de Pneumo-Phtisiologie” of Cotonou, Benin, little is known about the characteristics of patients who have not attended their scheduled appointment, the results of tracing and the possible benefits on improving treatment outcomes. This study aimed to determine the contribution of tracing activities for those who missed scheduled appointments towards a successful treatment outcome.

**Methods:**

A retrospective cohort study was carried out among all smear-positive pulmonary tuberculosis patients treated between January and September 2013. Data on demographic and diagnostic characteristics and treatment outcomes were accessed from tuberculosis registers and treatment cards. Information on those who missed their scheduled appointments was collected from the tracing tuberculosis register. A univariate analysis was performed to explore factors associated with missing a scheduled appointment.

**Results:**

Of 457 patients (410 new smear-positive and 47 retreatment tuberculosis), 37 (8 %) missed one or more of their appointments with a total of 44 episodes of missed appointments. The 3.5th (32 %) and 5th (43 %) month appointments were the ones most likely to be missed. Being male was associated with a higher risk of missing appointments (RR = 4.2; 95 % CI = 1.5–11.8, *p* = 0.004) while having HIV infection was associated with a lower risk (RR = 0.3, 95 % CI = 0.1–0.9, *p* = 0.03). Principal reasons for missed appointments were travelling outside Cotonou (34 %) and feeling better (21 %). In 24 (55 %) of these 44 episodes of missed appointments, contact was made with the patient who returned to the programme. These follow-up activities increased the treatment success by 4 %.

**Conclusion:**

In Cotonou, Benin, less than 10 % of tuberculosis patients miss at least one of their scheduled appointments. Tracing activities increase the treatment success rate by 4 % and current on-going practices in the Programme need to be endorsed and encouraged.

**Electronic supplementary material:**

The online version of this article (doi:10.1186/s12913-015-1219-z) contains supplementary material, which is available to authorized users.

## Background

Although treatment success rates in national tuberculosis control programmes (NTPs) have improved globally in the last few years to reach 86 % in 2013 [1], compliance with scheduled visits at clinics to collect drugs or submit sputum specimens for smear microscopy remains a serious concern. Poor attendance for follow-up visits compromises a successful treatment outcome and favours the emergence and subsequent transmission of drug resistant bacilli [2, 3]. Various reasons for poor attendance at clinics include patient-related factors, health care system failures and community beliefs [4].

To reduce this problem, NTP officers are encouraged to develop feasible strategies to prevent loss to follow-up and bring back to treatment those who have been declared loss-to-follow-up [2, 3, 5, 6]. The priority is to prevent losses to follow-up as significant efforts are required to trace and bring patients back to the programme.

Benin is a low-income country in West Africa. Since 2009, the country has achieved a treatment success of 90 % among new smear-positive TB cases, with approximately 1 % loss-to-follow-up [7, 8]. This excellent outcome is the result of several actions and strategies that are routinely implemented such as the quality of counselling before starting treatment, the application of Directly Observed Treatment (DOT) by health care workers and nutritional support. In addition, the NTP has established a special unit charged with tracing those who fail to attend DOT sessions or scheduled appointments (SA) during their ambulatory treatment. Currently, there is little reported information about the value of ensuring that SAs are kept. We therefore examined whether a focus on assisting patients who were irregular with their scheduled appointments was associated with improved treatment outcomes in Cotonou, the economic capital of Benin. Specific objectives were to determine in new and previously treated smear-positive pulmonary tuberculosis (TB) patients diagnosed and treated in the “Centre National Hospitalier de Pneumo-Phtisiologie” (CNHP-P) of Cotonou: i) the number (and proportion) who were traced for missing SA; ii) factors associated with missing SA; iii) results of the tracing; iv) reasons of missing the SA; and v) of those declared successfully treated, the number and proportion who were previously traced and the contribution of tracing activities to successful treatment outcomes.

## Methods

### Study design

This was a retrospective cohort study.

### Setting—general and study site

#### Country

Benin is a low-income country in West Africa with an estimated population of 10 million and a gross national income of US $890 per capita in 2014. (http://data.worldbank.org/country/benin). The national incidence rate of notified new TB cases has remained stable between 39 and 41 per 100,000 populations over the last 10 years [1].

#### NTP control and routine strategies to enhance treatment compliance

The Benin NTP follows the Direct Observed Treatment Strategy principles and uses recognised international criteria for the diagnosis and the treatment of patients with TB [2, 3, 9]. After the diagnosis of TB has been confirmed and before the start of treatment, the patient attends an education session, supervised by a medical practitioner and carried out by nurses in conjunction with social workers. Information is provided on the disease and therapy. Any concern that the patient may have is systematically sought and addressed as far as possible. In addition, patients are strongly recommended to provide one supporter with a complete address to help them through their treatment. Those who reside far from the Basic Management Unit (BMU) from where TB has been diagnosed are either referred to another BMU closer to the patient’s home or registered in the initial BMU and sent to a treatment clinic for DOT to avoid poor compliance resulting from poor health facility access.

TB patients are then either hospitalized or managed in an ambulatory fashion. During the intensive phase, DOT is strict and carried out daily by a nurse for all smear-positive (new and retreatment TB) cases. Every morning and before DOT is administered, patients receive an educational talk about some aspect of their disease and they also have the opportunity to discuss their concerns with social workers. Twice a month, a meeting is scheduled by the social services between the TB patients association and patients with newly diagnosed TB. After 2 months (or 3 months for retreatment TB cases), patients are once again briefed on how the continuation phase will be managed.

In the continuation phase of treatment, there is no DOT and patients are regularly supplied with medications for certain defined periods (between 30 and 45 days). At the time of medication collection, the patient is reminded about the next scheduled appointment by the nurse. Each patient also receives nutritional support at these visits for the whole duration of treatment.

In spite of all these measures, some patients may fail to attend their SA. For these patients, nurses in charge of the TB clinics can use a motor cycle and receive a monthly petrol allocation in order to trace these patients.

#### The CNHP-P and the “Service de relance”

The CNHP-P is the largest BMU and is situated in Cotonou, the economic capital of the country. This centre diagnoses and treats about 24 and 90 % of notified TB cases in Benin and Cotonou respectively.

The strategies that have been presented earlier to reduce poor treatment compliance are strictly implemented in CNHP-P. Tracing of patients who have not attended their SA is entirely in the hands of a unit in the centre (with four workers) named “Service de relance”. At the start of treatment, every TB patient is sent to the “Service de relance” in order to share proper contact details should there be a need for follow-up. Tracing is carried out about 5 days after patients miss their SA for DOT in the initial phase, for drug collection in the continuation phase and for sputum smear follow-up examination. The workers in the “service de relance” are informed by nurses about patient failures to attend SA, and tracing is carried out through phone calls and/or home visits sometimes with the help of the DOT supporter. A home service (sputum specimen collection or treatment delivery) can be carried out if necessary.

### Study participants

All smear-positive TB patients diagnosed and treated in the CNHP-P between 1st January and 30th September 2013 were included in the study.

### Data variables, source of data, definition of variables and data collection tool

Data collected from TB registers and treatment cards for each patient by included: TB registration number, demographic characteristics (sex, age, place of residence), type of TB (new smear-positive and retreatment TB), HIV-status (negative, positive, unknown), anti-tuberculosis treatment regimen and treatment outcomes.

For those who missed one (or more) of their SA, additional information was collected from the tracing TB register that included: timing of the missed SA, reasons and results of the tracing activity. The tracing TB register was filled in by workers of the “service de relance”. The information obtained was shared weekly and validated with nurses and the appropriate decisions were made for patients. These data were cross-checked with data on the treatment card to ensure that the patient really did miss a SA and the reason for missing the SA. All these data were collected by two investigators into a paper-based study questionnaire. Because of the retrospective nature of the study, other methods of validating the data were not possible.

### Analysis and statistics

Data from the questionnaires were double-entered into an electronic file using EpiData version 3.1 (EpiData Association, Odense, Denmark). Categorical variables were analyzed using frequencies and percentages. Factors associated with failing to attend the SA were identified and compared using the chi-square test, risk ratios (RR) and 95 % confidence intervals (95 % CI) as appropriate. Levels of significance were set at 5 %.

### Ethical considerations

Ethical approval for the study was obtained from the National TB Programme, Benin and the Ethics Advisory Group of the International Union Against Tuberculosis and Lung Disease, Paris, France. Because it was a retrospective review of already collected data, approval of the local ethics committee named “Comité National d’Ethique pour la Recherché en Santé” (http://www.ethique-sante.org/index.htm) was not required according to the country’s recommendations.

The need for informed consent was waived by the National TB Programme, Benin and the Ethics Advisory Group of the International Union Against Tuberculosis and Lung Disease, Paris, France.

## Results

There were 457 patients with smear-positive TB who started treatment at the CNHP-P (410 with new smear-positive TB and 47 with retreatment TB). The number and proportion of patients who failed to attend their SA before treatment completion and the types of SA missed are shown in Table 1. In total, there were 37 (8 %) patients who missed one or more of their SA. Of these, seven patients missed their SA on two occasions, making a total of 44 episodes of missed SA. In new smear-positive TB cases, the 3.5th and 5th month appointments were those most commonly missed in 32 and 43 % of the 44 episodes respectively. Two of these patients eventually had smear-positive sputum at the 5th month of follow-up and were declared failures to the first line treatment regimen, after their sputum had been collected during home visits - none was found with resistance to rifampicin.

**Table 1:** Scheduled appointments missed by tuberculosis patients diagnosed and treated in the CNHP-P, Cotonou, Benin, between January and September 2013

	All smear positive TB (%)	New smear positive TB (%)	Retreatment TB (Fraction)
Total TB patients	457	410	47
Patients who missed at least one SA^a^	37 (8)	36 (9)	1 (0.02)
Time at which patients missed their SA^b^			
In Initial phase before 2 months	6 (14)	6 (14)	0
At 2 months	5 (11)	5 (12)	0
At 3 months	0	-	0
At 3.5 months	14 (32)	14 (33)	-
At 5 months	19 (43)	18 (42)	1
Total appointments missed^c^	44	43	1

^a^The denominator is the total number of TB patients treated

^b^The denominator is the total number of appointments missed

^c^Some patients missed more than one scheduled appointment: three new smear-positive tuberculosis patients missed their scheduled appointments at 2 and 3.5 months; two missed at 3.5 and 5 months and two missed at the initial phase and at 5 months

Baseline characteristics associated with a missed SA during the treatment course are shown in Table 2. Being male was associated with a higher risk of missing a SA while having HIV infection was associated with a lower risk.

**Table 2:** Baseline characteristics associated with missed scheduled appointments in tuberculosis patients diagnosed and treated in the CNHP-P, Cotonou, Benin, between January and September 2013

Baseline characteristics		Missed scheduled appointments (%)	RR	95 % CI	*p* (value)
Sex	Female	4/153 (2.6)	1		
	Male	33/304 (10.9)	4.2	1.5–11.5	0.004
Age (years)	<25	5/82 (6.1)	1		
	25–64	31/360 (8.6)	1.4	0.6–3.5	0.21
	≥65	1/15 (6.7)	1.1	0.1–8.7	1
HIV-Status	Negative	34/346 (9.8)	1		
	Positive	3/108 (2.8)	0.3	0.1–0.9	0.03
	Unknown	0/3 (0)	-		-
Type of TB	New SS+	36/410 (9.8)	1		
	Retreatment	1/47 (4.3)	0.2	0.03–1.7	0.16
Place of treatment	CNHP-P	21/259 (8.1)	1		
	DOT clinic	16/198 (8.1)	1.0	0.5–1.9	0.99

*RR* Risk Ratio; *CI* Confidence Interval; *HIV* Human Immunodeficiency Virus; *SS+* New smear positive tuberculosis; *DOT* Directly Observed Treatment

All 37 patients with a missed SA were contacted by phone or visits by the “service de relance”. Results of further tracing activities are shown in Table 3. For 24 (55 %) of the 44 episodes of missed SA, the patients were contacted and either immediately returned to the centre or benefited from a home visit combined with the collection of their sputum. For the 20 episodes of missed SA for which there was no contact made with the patient, nine patients later returned to the centre on their free own will and completed their treatment. In these patients who could not be contacted, the contact address was correct in all but one patient, but the patients were not at home at the time of the visit.

**Table 3:** Results from tracing tuberculosis patients who missed their scheduled appointments while on treatment in the CNHP-P, Cotonou, Benin, between January and September 2013

Results		Number
Contact made with patient during the tracing		24
Results:	Immediate return to the centre	19
	Home service delivery	5
No contact made with patient during the tracing		20
Results:	Returned at some time to the centre on their own	9
Total missed appointments investigated		44

The reasons of missing the SA were known in 29 of the 44 episodes and are shown in Table 4. The two principal reasons were travelling outside the city of usual residence and feeling better on anti-tuberculosis treatment.

**Table 4:** Reasons of missing a scheduled appointment in tuberculosis patients diagnosed and treated in the CNHP-P, Cotonou, Benin, between January and September 2013

Known reasons for missing a scheduled appointment	Patients
Travel	10 (0.34)
Improving health condition	6 (0.21)
Death	3 (0.1)
Incarceration	2 (0.07)
Other^a^	8 (0.28)
Total^b^	29

^a^Other includes: Financial issues (1), Worsening conditions (1), lapse of memory (1), Hospitalization for comorbidity (1), psychiatric troubles (1), accident (1) attending a professional training course (1) and treatment resumption in another centre (1)

^b^Reasons of failing to attend the scheduled appointment were known for 29 out of 44 episodes

Of the 37 patients who had follow-up tracing undertaken, 26 completed their treatment successfully (17 were contacted during the tracing and 9 were not contacted but returned later to treatment). As shown in Table 5, the follow-up tracing activity increased the number of patients successfully treated in the centre by 4 %.

**Table 5:** Contribution of follow-up tracing activities in treatment success of tuberculosis patients treated in the CNHP-P, Cotonou, Benin, between January and September 2013

All smear positive tuberculosis patients	450
Patients with a successful outcome (%^a^)	403 (88)
Did not miss a scheduled appointment and did not need to be traced during treatment (%^b^)	377 (94)
Missed a scheduled appointment, traced but never contacted face-to-face and returned to treatment on their free own (%^b^)	9 (2)
Missed a scheduled appointment, traced and contacted face-to-face and brought back to treatment (%^b^)	17 (4)

^a^The percentage calculated from all patients (*N* = 450) who had been started on treatment

^b^The percentage calculated from all patients (*N* = 403) who were successfully treated

## Discussion

This is the first study in Benin within the NTP to assess the results and impact of tracing activities in patients with tuberculosis who missed their SA. In Cotonou, males were more likely to miss a SA while interestingly those with associated HIV infection were at lower risk. The SAs at 3.5 months and at 5 months were those most likely to be missed. Travel to a destination outside the city of residence and feeling better on anti-TB treatment were the two main factors associated with failing to attend the SA. We finally found that the tracing of those who failed to attend their SA, while not always successful in the face-to-face contact of patients, increased the treatment success rate by 4 % among patients treated in this city.

The strength of this study is that it was done within the context of the TB programme and included a large number of consecutively registered patients. In addition, the study report adhered to the Strengthening the Reporting of Observational Studies in Epidemiology (STROBE) guidelines (Additional file 1). The main limitation is that we only included patients from Cotonou and the results may not be representative of the country as a whole. Further limitations are the lack of a control group, limited information on baseline characteristics of participants and potential bias on data collection. Tracing was an already accepted component of the national programme and data routinely collected have been used for this study. These limitations cited above could be addressed in the future by a prospective cohort study that also will include a group control.

As a chronic disease, TB requires treatment for a minimum of 6 months. Thus, keeping patients with TB on treatment and ensuring that all SA are kept is not always easy and remains a permanent challenge for programmes [10, 11]. In routine practice, TB patients are usually traced if they are non compliant with treatment or miss their appointments either for sputum follow-up examination or collecting medication. Our study found that less than 10 % of TB patients on treatment were non-adherent. This proportion is lower than that reported from other settings in sub-Saharan Africa. For example, in one review of studies from Uganda, Ethiopia, and Zambia, the proportion of non-adherent TB patients ranged from 11.3 to 29.6 % [12]. In another review that included studies from Cape Town, Harare, Lusaka, Durban and Mbeya, overall more than one fourth of registered TB patients were non-adherent to treatment (26 %) [13]. In our study, we believe that the large majority of patients completed their treatment without needing any follow-up tracing because they understood the importance of regular, uninterrupted treatment to ensure cure of their disease, and this probably relates to good quality education from health care workers in the programme at the start of and during treatment. The most important factor in achieving this education is a strong willingness of health care workers to cure the patient and ensure good compliance of patients with scheduled appointments. This practice of persuading patients to fully comply with scheduled appointments is also regularly encouraged during supervisory visits, and in our view, this probably contributes to the low rate of loss-to-follow-up during TB treatment reported from the country [7]. In addition, in Cotonou, the overall good compliance with treatment is probably partly due to the assistance of patients who have already completed treatment, who thus share their experience and provide first-hand knowledge when it comes to patient support.

Despite this support, this study shows that some patients do not comply with their SA. This is more common in males which concurs with other reports from Africa and Asia [14, 15]. In contrast to our study, others have found that associated HIV infection is associated with poor compliance with treatment [11]. We are not surprised at our finding as most patients with HIV-associated TB in Benin are on antiretroviral therapy, which provides structured and monitored care and thus would tend to improve anti-TB treatment compliance. Missing the SA at 5 months is a concern because this is an opportunity to collect sputum specimens and identify treatment failure as early as possible [16], and one of the possible solutions is for the programme to collect sputum specimens at home in those who have not come for follow-up.

This study has several implications for the NTP. First, it is important that the Programme endorses and continues to encourage all the actions that prevent patients from being poorly adherent to treatment (i.e., the patients education, the health care workers’ motivation to providing good quality services, reminders at the different appointments, support of expert TB patients, food supplies and so on) and reaches and brings back to treatment those who are not adherent with their scheduled appointments. Second, more attention should be directed to male patients as they appear to be more likely to miss their SA. Third, there is a need to emphasize at the earlier 2 and 3 month appointments the importance of attending at 5 months in order for sputum smears to be checked at this time. Fourth, patients should be systematically asked whether they are likely to travel during treatment before the next SA, and if this is the case an extended supply of medications should be given to cover this period and advice given to redirect the patient to the nearest BMU for sputum smear examination. Finally, at the time of registration it is crucial to obtain a correct address, in case follow-up to the home is needed, and this study shows that in general this is done well in the programme in Cotonou.

## Conclusion

The proportion of TB patients who failed to attend a SA in Cotonou was less than that found in other settings in sub-Saharan Africa. When a SA is missed, the follow-up tracing activities result in a small, but important, increase in treatment success. Actions performed to prevent patients from discontinuing treatment and to trace non-adherent patients within the Programme need to continue so that Benin can maintain its good programme outcomes.

## Additional file

Additional file 1: **STROBE Statement—Checklist of items that should be included in reports of*****cohort studies.*** (PDF 18 kb)

## Abbreviations

NTP: National Tuberculosis Programme
TB: Tuberculosis
DOT: Directly Observed Treatment
SA: Scheduled Appointments
CNHP-P: “Centre National Hospitalier de Pneumo-Phtisiologie”
BMU: Basic Management Unit
HIV: Human Immunodeficiency Virus
